# Emergence of KPC-2 and NDM-5-coproducing hypervirulent carbapenem-resistant *Klebsiella pneumoniae* with high-risk sequence types ST11 and ST15

**DOI:** 10.1128/msphere.00612-23

**Published:** 2024-01-09

**Authors:** Ying Zhou, Xiaocui Wu, Chunyang Wu, Peiyao Zhou, Yang Yang, Bingjie Wang, Yanlei Xu, Huilin Zhao, Yinjuan Guo, Jingyi Yu, Fangyou Yu

**Affiliations:** 1 Department of Clinical Laboratory Medicine, Shanghai Pulmonary Hospital, Tongji University School of Medicine, Shanghai, China; 2 Department of Respiratory Medicine, The First Affiliated Hospital of Wenzhou Medical University, Wenzhou, China; 3 Department of Laboratory Medicine, The First Affiliated Hospital of Wenzhou Medical University, Wenzhou, China; 4 Institute of Antibiotics, Huashan Hospital, Fudan University, Shanghai, China; Hackensack Meridian Health Center for Discovery and Innovation, Nutley, New Jersey, USA

**Keywords:** *Klebsiella pneumoniae*, NDM-5, KPC-2, hypervirulent

## Abstract

The emergence of *Klebsiella pneumoniae* carbapenemase-2 (KPC-2) and New Delhi metallo-β-lactamase (NDM)-coproducing hypervirulent carbapenem-resistant *Klebsiella pneumoniae* (KPC-2-NDM-hv-CRKP) poses a certain threat to public health. Currently, only a few sporadic reports of such double-positive hv-CRKPs were available. In this study, we isolated two KPC-2-NDM-5-hv-CRKPs from elderly patients with serious underlying diseases and poor prognoses. We found both FK3122 and FK3127 were typical multidrug-resistant (MDR) isolates, exhibiting high-level resistance to both carbapenems and novel β-lactamase inhibitors ceftazidime/avibactam. Notably, FK3122 is even resistant to cefiderocol due to multiple *bla*_NDM-5_ elements. Besides the MDR phenotype, A549 human lung epithelial cells and *Galleria mellonella* infection model all indicated that FK3122 and FK3127 were highly pathogenic. According to the whole-genome sequencing analysis, we observed over 10 resistant elements, and the uncommon co-existence of *bla*_KPC-2_, *bla*_NDM-5_, and virulence plasmids in both two isolates. Both virulence plasmids identified in FK3122 and FK3127 shared a high identity with classical virulence plasmid pK2044, harboring specific hypervirulent factors: *rmpA* and *iuc* operon. We also found that the resistance and virulence plasmids in FK3127 could not only be transferred to *Escherichia coli* EC600 independently but also together as a co-transfer, which was additionally confirmed by the S1-pulsed-field gel electrophoresis plasmid profile. Moreover, polymorphic mobile genetic elements were found surrounding resistance genes, which may stimulate the mobilization of resistance genes and result in the duplication of these elements. Considering the combination of high pathogenicity, limited therapy options, and easy transmission of KPC-2-NDM-5-hv-CRKP, our study emphasizes the need for underscores the imperative for ongoing surveillance of these pathogens.

**IMPORTANCE:**

Hypervirulent *Klebsiella pneumoniae* drug resistance has increased gradually with the emergence of carbapenem-resistant hypervirulent *K. pneumoniae* (hv-CRKP). However, little information is available on the virulence characteristics of the New Delhi metallo-β-lactamase (NDM) and *Klebsiella pneumoniae* carbapenemase-2 (KPC-2) co-producing *K. pneumoniae* strains. In this study, we obtained two KPC-2-NDM-hv-CRKPs from elderly patients, each with distinct capsule types and sequence types: ST11-KL64 and ST15-KL24; these ST-type lineages are recognized as classical multidrug-resistant (MDR) *K. pneumoniae*. We found these KPC-2-NDM-hv-CRKPs were not only typical MDR isolates, including resistance to ceftazidime/avibactam and cefiderocol, but also displayed exceptionally high levels of pathogenicity. In addition, these high-risk factors can also be transferred to other isolates. Consequently, our study underscores the need for ongoing surveillance of these isolates due to their heightened pathogenicity, limited therapeutic options, and potential for easy transmission.

## INTRODUCTION

*Klebsiella pneumoniae* carbapenemase (KPC) and New Delhi metallo-β-lactamase (NDM), which are encoded by the *bla*_KPC_ and *bla*_NDM_ genes respectively, were two major carbapenemases in Enterobacteriaceae, especially in *Klebsiella pneumoniae* (1). These carbapenemases are capable of hydrolyzing not only carbapenems but also most other important β-lactam antibiotics, making them a significant challenge to clinical treatment (2). Carbapenem-resistant *K. pneumoniae* (CRKP) was listed as the most urgent antibiotic resistance threat (1). On the other hand, a different type of *Klebsiella pneumoniae* known as hypervirulent *K. pneumoniae* (hvKP) mainly infects healthy individuals and can cause severe disseminated infections (3). Recently, an increasing number of *K. pneumoniae* strains have been identified that combine hypervirulent and multidrug-resistant characteristics, referred to as hv-CRKP (4). However, the hypervirulent *K. pneumoniae* strains that co-produce NDM and KPC-2 carbapenemases were rare to find.

In China mainland, only five KPC-2 and NDM co-producing hv-CRKP isolates (KPC-2-NDM-hv-CRKPs) have been identified with different sequence types (ST11, ST86, and ST464) (5–9). However, as such KPC-2-NDM-hv-CRKPs were rare, a complete understanding of these double-positive hv-CRKPs may not be adequately described. In this study, we obtained two KPC-2-NDM-hv-CRKPs with different capsule and sequence types, ST11-KL64 and ST15-KL24. Specifically, ST11 and ST15 type isolates were typically identified as classical CRKP. Previous multicenter epidemiological study in China, also indicated that the KPC-2-NDM-CRKPs were mainly typed as ST11 and ST15, but the hypervirulent phenotypes were absent in these strains (2). The genetic features and epidemic characteristics of KPC-2-NDM-hv-CRKP infections are still unidentified. These are critical questions that must be answered because the combination of "such double-super-antimicrobial resistance and hypervirulence" may significantly increase the "superbug’s" public health threat.

In this study, we not only featured the antibiotic-resistant and hypervirulent phenotypes in detail, but we also investigated the molecular mechanisms associated with multidrug resistance and hypervirulent phenotypes using whole-genome sequencing (WGS). We also analyzed the plasmid-backbone and conjugation modules and used a conjugation assay to further determine the self-transmissibility of these resistance and hypervirulence genes to study how these isolates emerged and the risk of dissemination of these isolates. According to our findings, the KPC-2-NDM-hv-CRKP poses a significant threat to healthcare networks, and steps should be taken to closely monitor and control the spread of superbugs with multidrug-resistant phenotypes and hypervirulence.

## MATERIALS AND METHODS

### Bacterial strains

Both *K. pneumoniae* strains FK3122 and FK3127 were isolated from elderly patients with serious underlying diseases and poor prognosis. The patient infected with the FK3122 was diagnosed with severe encephalic infection and the patient infected with the FK3127 was diagnosed with severe pulmonary infection. Both strains exhibited typical hypervirulent and carbapenem-resistant phenotypes. *K. pneumoniae* strain 1627 (classical *K. pneumoniae*, cKp, ST11) and FK3036 (classical *K. pneumoniae*, cKp, ST15) were used as virulence-negative control strains, and NUTH-K2044 (ST23-KL1) (10) was used as a virulence-positive control strain.

### Antimicrobial susceptibility test

According to the Clinical and Laboratory Standards Institute guidelines, the minimal inhibitory concentration (MIC) of the *K. pneumoniae* strains FK3122 and FK3127 was determined by broth microdilution. We used *Escherichia coli* ATCC 25922 as a quality control strain for determining MICs. The interpretative breakpoints were based on CLSI2022-M100-ED31. The cefiderocol breakpoints were based on EUCAST 2022.

### WGS and bioinformatics analysis

The genomic DNA of FK3122 and FK3127 was extracted with a commercial kit and sequenced using PacBio Sequel and Illumina NovaSeq 6000. Reads were filtered and assembled using SPAdes (version 3.12) (11), A5-miseq (12), and CANU software (version 1.7.1) (13). Kleborate (https://github.com/katholt/Kleborate/) and PlasmidFinder databases (https://cge.cbs.dtu.dk/services/PlasmidFinder/) were used to determine Multilocus sequence typing (MLST), serotype, resistance, and virulence determinants, as well as plasmid replicons (14). Conjugation modules were analyzed using oriTfinder (https://tool-mml.sjtu.edu.cn/oriTfinder/oriTfinder.html), and circular plasmid maps were generated using CGviewer.

### Quantitative siderophore production assay

To assess the ability of bacterial supernatants to chelate iron, the researchers utilized the chrome azurol S (CAS) assay, following established protocols (15). Specifically, 10 mL of stationary-phase, iron-chelated cultures was deposited on CAS plates, and after 48 hours of incubation at 37°C, the presence of orange halos was used to identify siderophore production.

### Cell culture and *K. pneumoniae* infection

A549 lung cells (ATCC CCL-185) were cultured in Dulbecco’s modified Eagle’s medium containing 10% fetal bovine serum in a 5% CO_2_ incubator. Cells were seeded in 24-well plates at a density of 1 × 10^5^ cells per well and incubated for 24 hours. The cell-culture medium was then supplemented with 2 × 10^7^ CFU of *K. pneumoniae* and incubated for 21 hours. After centrifugation at 3,000 rpm and 4°C for 5 minutes, the supernatant was analyzed for lactate dehydrogenase (LDH) using the LDH Cytotoxicity Assay kit (Solarbio BC0685), following the manufacturer’s instructions.

### *G. mellonella in vivo* infection model

To assess the pathogenicity of *K. pneumoniae* strains FK3122 and FK3127, we conducted *G. mellonella* infection assays. Caterpillars weighing between 150 and 200 mg were selected after storage at 4°C. Treatment groups were inoculated with 10 µL of the bacterial suspension containing 1 × 10^6^ CFU/mL, while the control group received normal saline. Each treatment group had at least 30 caterpillars, divided into three Petri dishes, and maintained at 37°C. Survival rates were recorded for 4 days with daily observations.

### Conjugation assay

We used a conjugation assay to test if the resistant plasmids from *K. pneumoniae* FK3122 and FK3127 could be transferred or co-transferred to *E. coli* EC600 (recipient isolate). Donors and recipients were cultured to logarithmic phase, mixed in a 1:1 ratio, centrifuged at 8,000*g* for 1 minute, and resuspended in 20 µL of 10 mM MgSO_4_. The resuspension was plated on LB agar and incubated at 37°C overnight. The number of transconjugants per donor was calculated to determine the conjugation frequency. PCR was performed to confirm the presence of the *bla*_KPC-2_, *bla*_NDM-5_, and *iuc* genes in all transconjugants, using primers listed in Table S1.

### S1-pulsed-field gel electrophoresis assay

To confirm plasmid presence in *K. pneumoniae* FK3127 and its transconjugants, S1-pulsed-field gel electrophoresis (S1-PFGE) was used. All PFGE plugs were prepared and digested using a previously described method, then separated for 19 hours on a 1.0% agarose gel using XbaI-digested Salmonella H9812 DNA as a marker. The CHEF mapper system (Bio-Rad) was used for PFGE analysis, and DNA in the gels was stained with Gel-red (Yeasen, China) at 6 V/cm and 14°C with pulse times ranging from 4 to 40 seconds. S1 nuclease endonuclease (Takara, Dalian, China) was used to treat the isolates, which were embedded in 10 g/L Seakem Gold gel before PFGE analysis.

### Nucleotide sequence accession numbers of *K. pneumoniae* FK3122 and FK3127

The complete nucleotide sequences of the *K. pneumoniae* FK3122 and FK3127 were submitted to GenBank under accession numbers JAOVSH000000000 and JAOVSI000000000, respectively.

### Statistics

Statistical significance was assessed using a two-tailed Student’s *t*-test and log-rank (Mantel–Cox) test of the GraphPad Prism9 software. *P* < 0.05 was considered statistically significant.

## RESULTS

### *K. pneumoniae* FK3122 and FK3127 exhibited multidrug resistance phenotype

According to the antibiotic susceptibility test, we discovered that *K. pneumoniae* FK3122 and FK3127 exhibited similar multidrug-resistant profiles (Table 1); they revealed extensive resistance to all β-lactam antibiotics, including carbapenems, as well as the new β-lactamase inhibitors ceftazidime/avibactam (CZA). Notably, in addition to being resistant to ceftazidime/avibactam, the FK3122 was even resistant to cefiderocol, both of which were recognized as the new effective agents to defend the multidrug-resistant isolates (Table 1). Moreover, the antibiotics that can act against the infection caused by FK3127 were also limited, since it also exhibited high-level resistance toward aminoglycoside antibiotics. These antibiotic-resistant phenotypes were threatened for clinical treatments.

**TABLE 1:** Antimicrobial drug susceptibility profiles^*a*^

Antibiotics	MIC (mg/L)/antimicrobial susceptibility						
	Strains			Transconjugants			
	K.pneumoniae FK3122	K.pneumoniae FK3127	E.coli EC600	p3127-6-EC600(Virulence plasmid)	p3127-7-EC600(*bla*_KPC-2_ plasmid)	p3127-8-EC600(*bla*_NDM-5_ plasmid)	p3127-6&8-EC600(Virulence&*bla*_NDM-5_ plasmid plasmids)
MEM	>16/R	>16/R	<=0.5/S	<=0.5/S	8 /R	>16/R	>16/R
IPM	>16/R	>16/R	<=0.5/S	<=0.5/S	8 /R	>16/R	>16/R
GEN	<=0.5/S	16 /R	<=0.5/S	<=0.5/S	<=0.5/S	16 /R	16 /R
AMK	<=2/S	>64/R	<=2/S	<=2/S	<=2/S	>64/R	>64/R
ATM/AVI	<=1/4 /S	<=1/4 /S	<=1/4 /S	<=1/4 /S	<=1/4 /S	<=1/4 /S	<=1/4 /S
CZA	>32/4 /R	>32/4 /R	<=0.25/4	<=0.25/4	<=0.25/4	>32/4 /R	>32/4 /R
FDC	4 /R	0.5 /S	0.5 /S	0.5 /S	0.5 /S	0.5 /S	0.5 /S
PMB	1 /S	<=0.5/S	1 /S	1 /S	1 /S	1 /S	1 /S
AMP	>4/R	>4/R	<=0.5/S	<=0.5/S	>4/R	>4/R	>4/R
CAZ	>32/R	>32/R	<=1/S	<=1/S	>32/R	>32/R	>32/R
FEP	>16/R	>16/R	<=0.5/S	<=0.5/S	>16/R	>16/R	>16/R
FOX	>32/R	>32/R	4 /S	4 /S	>32/R	>32/R	>32/R
CTX	>64/R	>64/R	<=1/S	<=1/S	>64/R	>64/R	>64/R
TZP	>128/4 /R	>128/4 /R	<=4/4 /S	<=4/4 /S	>128/4 /R	>128/4 /R	>128/4 /R
CIP	>4/R	>4/R	<=0.25/S	<=0.25/S	0.5 /S	<=0.25/S	<=0.25/S
TCY	>16/R	4 /S	<=1/S	<=1/S	<=1/S	<=1/S	<=1/S
ATM	>32/R	>32/R	<=1/S	<=1/S	>32/R	<=1/S	<=1/S
SXT	>2/38 /R	>2/38 /R	<=0.5/9.5 /S	<=0.5/9.5 /S	<=0.5/9.5 /S	>2/38 /R	>2/38 /R
TGC	2 /S	0.5 /S	<=0.25/S	<=0.25/S	<=0.25/S	<=0.25/S	<=0.25/S

^*a*^ MIC, minimal inhibitory concentration; MEM, meropenem; IPM, imipenem; GEN, gentamicin; AMK, amikacin; ATM/AVI, aztreonam-avibactam; CZA, ceftazidime-avibactam; FDC, cefiderocol; PMB, polymyxin B; AMP, ampicillin; CAZ, ceftazidime; FEP, cefepime; FOX, cefoxitin; CTX, cefotaxime; TZP, piperacillin-tazobactam; CIP, ciprofloxacin; TCY, tetracycline; ATM, aztreonam; SXT, trimethoprim/sulfamethoxazole; and TGC, tigecycline.

### *K. pneumoniae* FK3122 and FK3127 revealed hypervirulent phenotype

**Fig 1:**
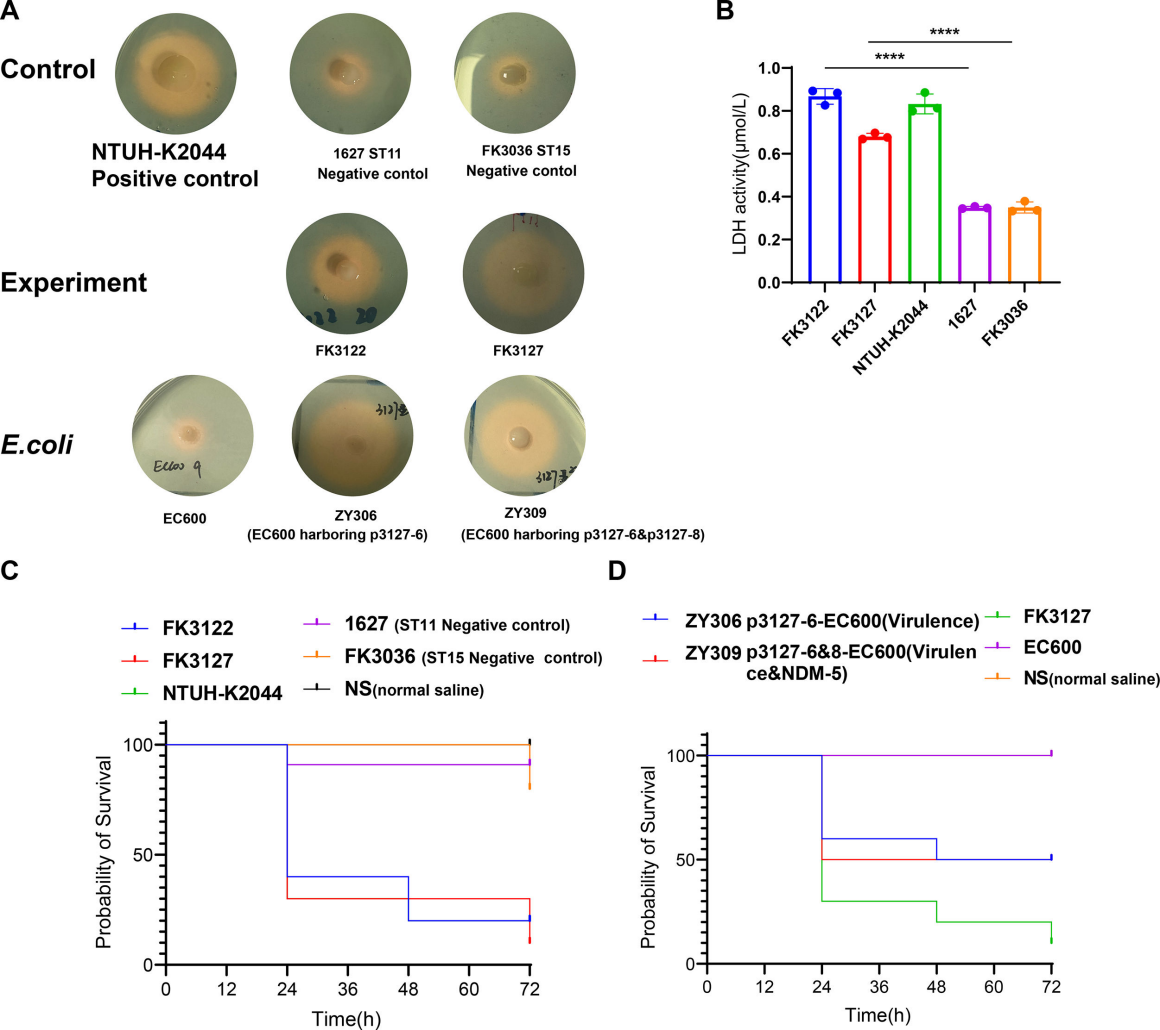
The virulence phenotypes and levels of FK3122 and FK3127. (**A**) Siderophores production determined by CAS agar plate. (**B**) The LDH activity in A549 human lung epithelial cells infected by different strains. (**C**) The survival curves of *G. mellonella* infected by FK3122, FK3127, and other control strains. (**D**) The survival curves of *G. mellonella* infected by different conjugants (*E. coli*). Note: 1627 (ST11 cKP, negative control), FK3036 (ST15 cKP, negative control), NS (normal saline). Unpaired two-sided Student’s *t*-test was performed for LDH production. A log-rank (Mantel–Cox) test was performed for the survival curves. ****P < 0.0001.

Here, we aimed to explore whether FK3122 and FK3127 pose hypervirulent features. The negative string test results indicated that the hypermucoviscous phenotypes were absent in FK3122 and FK3127. Quantitative siderophore assays indicated that FK3122 (ST11) and FK3127 (ST15) produced comparable siderophores with NTUH-K2044 (ST23, positive control), and produced substantially more siderophores than 1627 (ST11 cKP, negative control) and FK3036 (ST15 cKP, negative control) strains (Fig. 1A). *In vitro,* infection of A549 human lung epithelial cells was used to assess the virulency capacity of these isolates. FK3122 and FK3127 significantly increased the LDH activity in A549 cells compared to virulence-negative ST11 (1627, 0.826 µmol/L vs 0.355 µmol/L) and ST15 controls (0.697 µmol/L vs 0.349 µmol/L). Notably, both two strains showed comparable LDH release with the hypervirulent NTUH-K2044 (Fig. 1B). Furthermore, we applied *G. mellonella* larvae infecting model to calculate pathogenicity. At 3-day post-infection, the FK3122 (ST11, 20%), FK3127 (ST15, 10%), and NTUH-K2044 (positive control, 10%) showed comparable virulence resulting in 10%–20% survival. Notably, survival of a classic ST11cKP (1627, 90%) and ST15 cKP (FK3036, 80%) isolate reached 80%–90%, suggesting a high level of pathogenicity of FK3122 and FK3127 (Fig. 1C).

### *K. pneumoniae* FK3122 and FK3127 co-harboring *bla*_KPC-2_, *bla*_NDM-5_, and hypervirulent plasmids

We found that FK3122 belonged to ST11-KL64 isolates and FK3127 belonged to ST15-KL24 isolates, both ST-type lineages in which they belong were associated with the CRKP epidemic. In these isolates, we discovered more than 10 resistant elements as well as several key resistance plasmids (Table 2). The co-existence of *bla*_KPC-2_ and *bla*_NDM-5_ was studied because it played a pivotal role in conferring resistance to carbapenems and ceftazidime/avibactam. Notably, in the FK3122, we observed four copies of *bla*_NDM-5_, located in the chromosome, and two plasmids, respectively, which may account for its resistance to cefiderocol. Moreover, the presence of *rmtB* in FK3127 conferred higher-level resistance to aminoglycoside antibiotics (Table 2). Importantly, most of the resistance genes were carried by plasmids and were flanked by numerous mobile elements, heightening the risk of transmission. The *bla*_KPC_ gene was carried by the classical Tn*1721* transposon (16)*,* and the *rmtB* was surrounded by the IS*26* islands (“IS*26*-Tn*3*-IS*6100-mph(A*)-IS*26*-Tn*3-bla*_TEM-1B_-*rmtB*”), which was different from previous findings (17).

**TABLE 2:** General features and antimicrobial resistance genes of plasmids in *K. pneumoniae* FK3122 and FK3127

Characteristics		FK3122 ST11-KL64				FK3127 ST15-KL24		
	Chromosome	p3122-5	p3122-6	p3122-10	p3122-11	p3127-6	p3127-7	p3127-8
Length (bp)	/	184,935	80,896	112,567	19,905	197,626	95,828	91,040
GC content (%)	/	50	55	54	57	47	53	53
No. of ORF^*a*^	/	235	133	139	25	213	132	127
Incapability group	/	IncHI1B (pNDM-MAR), repB	IncFII (pHN7A8), IncR	IncFII (pCRY)		IncFIB (pNDM-Mar)	IncFII (pBK30683)	IncFII
Mobile ability	/	No	No	Yes	No	Yes	Yes	Yes
*OriT* (start…stop) (bp)	/	/	/	42,472…42,553		21,747…21,774	61,532…61,588	62,598…62,683
Relaxase (start…stop) (bp)	/	/	/	42,909…44,837		99,350…102,298	33,007…33,687	29,843…32,242
T4CP (start…stop) (bp)	/	168,995…170,194	/	73,597…75,789		102,288…104,414	33,687…35,999	35,313…37,175
T4SS (start…stop) (bp)	/	/	/	50,696…75,789		82,030…104,414	27,661…62,146	29,077…63,252
	/					170,586…197,624		
Resistant genes	*tet(A*)	/	***bla*_KPC-2_**	*bla*_NDM-5_ *2	***bla*_NDM-5_**		***bla*_KPC-2_**	***bla*_NDM-5_**
	*bla*_NDM-5_			*bla*_LAP-2_	*sul1*			*bla*_TEM-1B_
	*sul1*			*dfrA12*	*qacE*			*rmtB*
	*qacE*			*sul1*	*aadA2*			*aadA2*
	*aadA2*			*qacE*	*dfrA12*			*sul1*
	*dfrA12*			*aadA2*	*tet(A*)			*dfrA12*
				*tet(A*)				*mph(A*)
				*qnrS1*				
				*dfrA14*				
				*sul2*				
				*catA2*				
								
Virulence factors		***iucABCD/iutA^b^***				***iucABCD/iutA***		
		***rmpA/rmpA2***				***rmpA***		
		***iroN***						

^*a*^ ORF: Open reading frame.

^*b*^ These bolded resistance genes and virulence genes are very important.

**Fig 2:**
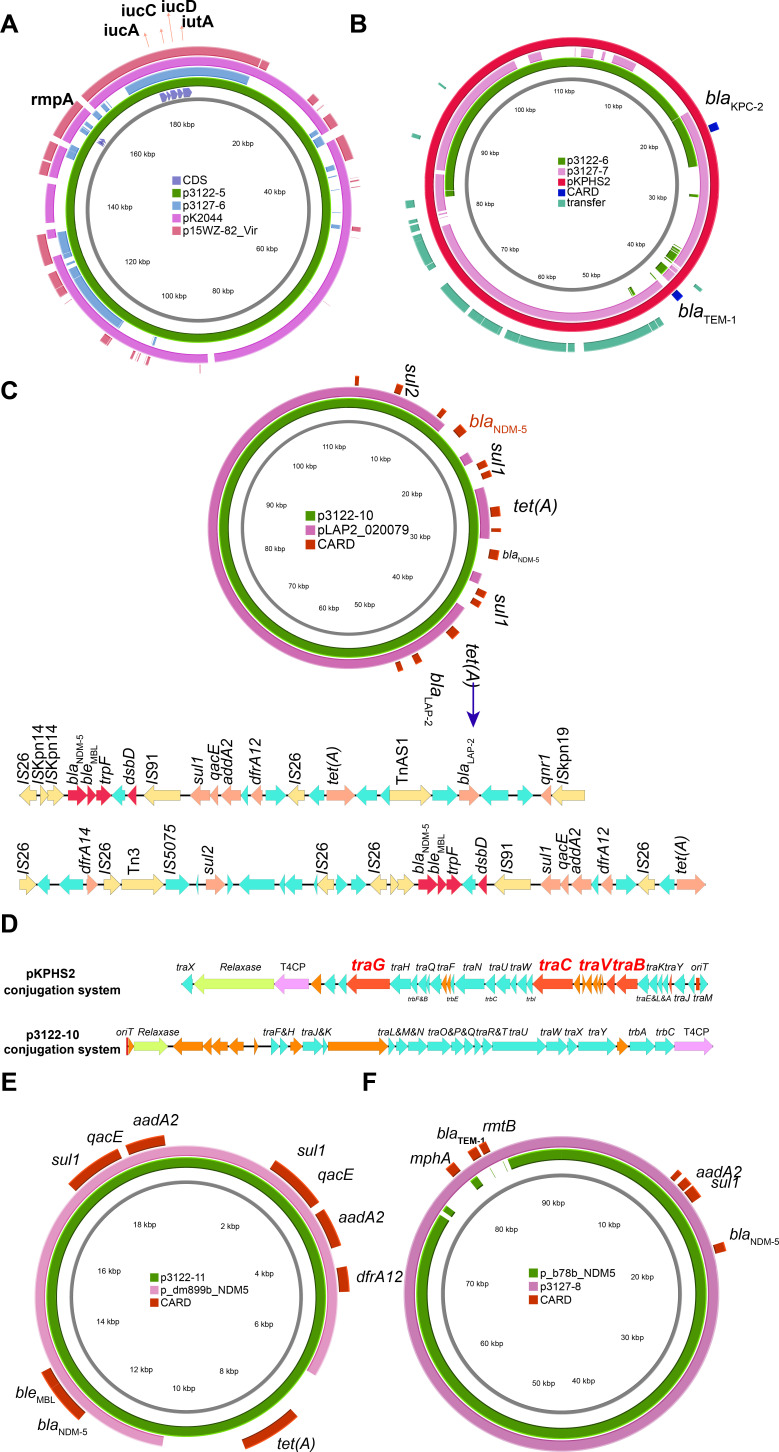
Comparative analysis of *bla*_KPC-2_, *bla*_NDM-5_, and virulence plasmids in FK3122 and FK3127. (**A**) Genome alignment was performed with virulence plasmids p3122-5 (JAOVSH010000005.1), p3127-6 (JAOVSI010000006.1), and with classical virulent plasmid pK2044 (CP026012.1), and conjugative virulence plasmid p15WZ-82_Vir (CP032356.1). (**B**) Genome alignment was performed with *bla*_KPC-2_ plasmids p3122-6 (JAOVSH010000006.1), p3127-7 (JAOVSI010000007.1), and with classical *bla*_KPC-2_ plasmid pKPHS2 (CP003224.1). (**C**) Genome alignment was performed with p3122-10 (JAOVSH010000010.1) harboring two copy *bla*_NDM-5_ elements with pLAP2_020079 (CP029382.1), and the structure of the two copy *bla*_NDM-5_ elements in p3122-10. (**D**) Comparison of the conjugative system of the reference plasmid pKPHS2 (CP003224.1) and p3122-10. (**E**) Genome alignment was performed with *bla*_NDM-5_ plasmid p3122-11 (JAOVSH010000011.1) with p_dm899b_NDM5 (CP095663.1). (**F**) Genome alignment was performed with *bla*_NDM-5_ plasmid p3127-8 (JAOVSI010000008.1) with p_b78b_NDM5 (CP095588.1).

In the two isolates, we observed two virulence plasmids, harboring specific virulent factors: *rmpA* and *iuc* operon (Table 2). In FK3122, p3122-5 was the virulence plasmid, typed as IncHI1B, shared high identity (coverage 93%, identity 99.86%) with conventional virulence plasmid pK2044 (CP026012.1) (Fig. 2A). Although we could observe the type IV coupling protein (T4CP) in p3122-5, the absence of the *oriT* (origin of transfer site) sites makes it lose the mobilizability. In FK3127, the virulence plasmid p3127-6 was different from the p3122-5, which was typed as IncFIB and harbored four complete conjugation modules. p3127-6 was a typical hybrid conjugative virulence plasmid, which shared approximately 100 kb of overlapping sequences that contained the virulence genes with pK2044 and p15WZ-82_Vir (18, 19) (CP032356.1, IncFIB, conjugative plasmid）(Fig. 2A). The virulence genes were found in two distinct regions of the 100 kb fragment, each flanked by transposable elements.

### Polymorphic mobile genetic elements contributed to the generation of the multiple copies of *bla*_NDM-5_ in FK3122

We observed three resistant plasmids in FK3122, p3122-6 (*bla*_KPC-2_), p3122-10 (two copies of *bla*_NDM-5_), and p3122-11 (*bla*_NDM-5_) (Table 2; Fig. 2). Both p3122-6 (IncFII) and p3122-11 were typed as non-mobilizable plasmids since they lose the essential trans-conjugative elements. When compared with the typical IncFII *bla*_KPC-2_ plasmid pKPHS2 (CP003224.1), the p3122-6 shared approximately 50% overlapping sequences, while the conjugation modules were absent (Fig. 2B). Notably, despite the presence of conjugative modules, the p3122-10 plasmid did not transfer to the EC600 recipients. When compared to the conjugative system of pKPHS2, we revealed that the system in p3122-10 lacked four important type 4 secretion system (T4SS)-associated proteins, TraB, TraC, TraG, and TraV, which contribute significantly to F pilus assembly (20) (Fig. 2D). The lack of crucial functioning proteins may explain why the plasmid p3122-10 does not self-transmit.

Although the plasmids identified in the FK3122 seemed less transmissible, the movement of insertion sequence IS*26* surrounding the *bla*_NDM-5_ genes was active. IS*26* is important in the spread of antibiotic resistance genes in Gram-negative bacteria, forming regions with multiple antibiotic resistance genes flanked by and interspersed with copies of IS*26* (21). In FK3122, we observed four copies of *bla*_NDM-5_ genes. The conservative *bla*_NDM-5_ resistant islands were “IS*26-bla*_NDM-5_-*ble*_MBL_-*trpF-dsbD- IS*CR1-*sul1-qacE-addA2-dfrA12*-IS*26*” (Fig. 2C). The duplication of *bla*_NDM-5_ was produced by an IS*26*-formed composite transposon, which was presumably activated by *IS*CR1, which uses a rolling-circle mechanism and can promote tandem duplication by homologous recombination (21). Moreover, the Tn*3*-derived inverted-repeat transposable elements were also been found to mobilize *bla*_NDM-5_. These results suggested that mobile genetic elements played an important role in the mobilization of *bla*_NDM-5_.

### Hypervirulent and carbapenems-resistant phenotype could be transferred from FK3127 to other isolates

Three key virulence and resistance plasmids in FK3127 were all recognized as conjugative plasmids (Table 2; Fig. 2). p3127-6 was a typical hybrid conjugative virulence plasmid, which could be effectively transferred to *E. coli* EC600 (0.52 × 10^−6^ to 4.9 × 10^−6^), and increased the pathogenicity of the recipients (Table 3; Fig. 1). We detected the siderophore production of EC600 and ZY306 (EC600 harboring p3127-6 virulence plasmid) and observed the remarkable siderophore production increase in ZY306 (Fig. 1A). Moreover, in the *G. mellonella* infection model, obtaining p3127-6 virulence plasmid significantly increased the mortalities in comparison to the *E. coli* EC600 recipients (Fig. 1D). p3127-7 (*bla*_KPC-2_) (0.9 × 10^−8^ to 2.4 × 10^−8^) were typed as IncFII (pBK30683), and p3127-8 (*bla*_NDM-5_ and *rmtB*) (0.8 × 10^−8^ to 4.9 × 10^−8^) were typed as IncFII conjugative plasmids. We found these resistance phenotypes could be transferred to the recipients through the plasmids. Notably, not only was the resistance phenotype delivered to the recipients, but also the virulence. We observed that the p3127-8 (*bla*_NDM-5_ and *rmtB*) and p3127-6 (virulence) plasmids could be co-transferred to the *E. coli* EC600 recipients (1.13 × 10^−8^ to 1.9 × 10^−8^), and the siderophore production and *G. mellonella* infection model also confirmed that (Table 3, Fig. 1; Fig. S1). Taken together, three plasmids identified in FK3127 not only endowed FK3127 with a hypervirulent and resistant phenotype but also can spread this high-risk phenotype to other strains, which is worthy of attention.

**TABLE 3:** Conjugation frequency of resistant plasmids identified in *K. pneumoniae* FK3127

Plasmid	Resistance or virulence gene	No. of independent determinations	Conjugation frequencies	
			Mean	Range
p3127-6	*iucABCD/iutA*	3	2.51 × 10^−6^	0.52 × 10^−6^ to 4.9 × 10^−6^
p3127-7	*bla*_KPC-2_	3	1.65 × 10^−8^	0.9 × 10^−8^ to 2.4 × 10^−8^
p3127-8	*bla*_NDM-5_	3	2.27 × 10^−8^	0.8 × 10^−8^ to 4.9 × 10^−8^
Co-transfer of plasmids				
Co-transfer of p3127-6 and p3127-8	*bla*_NDM-5_ + *iucABCD/iutA*	3	2.25 × 10^−8^	1.13 × 10^−8^ to 1.9 × 10^−8^

## DISCUSSION

The emergency of hypervirulent carbapenem-resistant *Klebsiella pneumoniae* (hv-CRKP) poses devastating challenges to public health. Of the epidemiology, most hv-CRKPs were associated with the *bla*_KPC-2_ genes and ST11-KL64 isolates (22).To date, only a few occasional reports of strains co-producing NDM and KPC-2 are available. When *K. pneumoniae* strains combine hypervirulence and carbapenem resistance, clinical treatment becomes complex, and available antibiotics are limited. When hv-CRKP co-harbors NDM and KPC-2, the treatment outlook becomes even worse, as CZA is rendered ineffective due to NDM carbapenemase production. In this study, we characterized two hv-CRKPs that co-harbored NDM, KPC-2, and virulence plasmid. Notably, these isolates derive from two pandemic CRKP lineages, ST11 and ST15.

Epidemiological data indicated that ST11-KL64-hvCRKP has emerged as the most prevalent hypervirulent and carbapenem-resistant *K. pneumoniae,* and usually contributed to the hospital outbreaks of infection (4, 22). FK3122 was a typical ST11-KL64-hvCRKP with the presence of IncHI1B virulence plasmid p3122-5 and IncFII *bla*_KPC-2_ plasmid p3122-6. However, unlike the classical IncFII *bla*_KPC-2_ plasmid and IncHI1B virulence plasmid, both two plasmids were identified to lose the mobile ability. In addition to the *bla*_KPC-2_ plasmid and virulence plasmid, we also observed four copies of *bla*_NDM-5_ genes, located in two plasmids and chromosomes. Notably, though plasmid p3122-10 (*bla*_NDM-5_) harbored conjugation systems, it could not be self-transferred effectively, since it lost the key protein to form the membrane-associated mating pair formation complex (20). Although both two *bla*_NDM-5_ plasmids in FK3122 lose effective transmissibility, the duplication transposon mobile of *bla*_NDM-5_ genes should attract attention. Previous studies have demonstrated that increased copy numbers of *bla*_NDM-5_ genes through translocation events would contribute to cefiderocol resistance (23). In our study, we also observed the increased cefiderocol resistance level of FK3122 (four *bla*_NDM-5_ copies) compared to the FK3127 (single *bla*_NDM-5_ copy). Moreover, the co-occurrence of *bla*_NDM-5_, *bla*_KPC-2_, and virulence plasmids was also identified in another ST11-KL64 strain KPWX136 in east China, but which did not exhibit the high pathogenicity as the isolate obtained in this study (5).

ST15 *K. pneumoniae* was identified as the second most frequent CRKP clone in hospital infections after ST11 *K. pneumoniae*, while the ST15 hypervirulent CRKP was uncommon (1). At present, there were only a few hypervirulent, and carbapenem-resistant ST15 *Klebsiella pneumoniae* have been described, most were associated with *bla*_OXA-232_ and *bla*_KPC-2_ (24, 25). In FK3127, we observed three key plasmids, p3127-6 (virulence), p3217-7(*bla*_KPC-2_), and p3127-8(*bla*_NDM-5_). Notably, all these plasmids not only could self-transfer to other isolates but also could co-transfer. Previous reports of cases of NDM-KPC-2-hv-CRKP infection are scarce, and no study found that the resistance determinants (*bla*_NDM_ and *bla*_KPC_) and virulence determinants all could be transferred in one isolate or even co-transferred. p3127-6 virulence plasmid was a typical hybrid conjugative plasmid. Previous genomic research indicated that these hybrid plasmids were most likely formed by the recombination of IncF and the virulence IncHIB plasmids (26); this hybrid pattern also was confirmed in the p3127-6 plasmid. p3127-7 and p3127-8 were all typical IncFII plasmids with completed conjugation modules and effective conjugation frequencies. The co-existence of virulence plasmid and resistance plasmids facilitated the formation of hv-CPKP. Moreover, the co-existence of transmissible resistance and virulence plasmids in one isolate could act as a vast storage pool of resistant and virulence elements. All in all, the ST15 FK3127 posed a major threat to modern medicine, jeopardizing our ability to treat life-threatening infections.

In this study, two high-risk novel *K. pneumoniae* co-harboring *bla*_NDM-5_, *bla*_KPC-2_, and virulence plasmids were characterized. Phenotype testing and genome background analysis confirmed the increased virulence and multidrug resistance of these strains. The superbug, which combines hypervirulence and carbapenem resistance, will endanger clinical treatment. To prevent widespread transmission, tailored surveillance, antimicrobial stewardship, and strict clinical management are urgently required.

## SUPPLEMENTAL MATERIAL

Additional experimental details msphere.00612-23-s0001.docx Some information about strains and several additional results.

ASM does not own the copyrights to Supplemental Material that may be linked to, or accessed through, an article. The authors have granted ASM a non-exclusive, world-wide license to publish the Supplemental Material files. Please contact the corresponding author directly for reuse.
