# Supplementary material for: Emergence of KPC-2 and NDM-5-coproducing hypervirulent carbapenem-resistant Klebsiella pneumoniae with high-risk sequence types ST11 and ST15
Source: mSphere. 2024 Jan 9;9(1):e00612-23. doi: 10.1128/msphere.00612-23 (PMC10826354; doi:10.1128/msphere.00612-23)
Supplement: Additional experimental details — Some information about strains and several additional results. [file msphere.00612-23-s0001.docx]

**Table S1 Strains and primers used in this study**

| **Strains** | **Relevant characteristics** | **Source or reference** |
| --- | --- | --- |
| ZY306 | *E. coli* EC600 harboring p3127-6 (virulecne plasmid in FK3127) | This study |
| ZY307 | *E. coli* EC600 harboring p3127-7 (*bla*_KPC-2_ plasmid in FK3127) | This study |
| ZY308 | *E. coli* EC600 harboring p3127-8 (*bla*_NDM-5_ plasmid in FK3127) | This study |
| ZY309 | *E. coli* EC600 co-harboring p3127-6 and p3127-8 (virulence plasmid and *bla*_NDM-5_ plasmid in FK3127) | This study |
| **Primers** |  |  |
| Name | Sequence |  |
| *bla*_KPC_-F | TCGCTAAACTCGAACAGG |  |
| *bla*_KPC_-R | TTACTGCCCGTTGACGCCCAATCC |  |
| *bla*_NDM_-F | GTCTGGCAGCACACTTCCTA |  |
| *bla*_NDM_-R | GCGGGCCGTATGAGTGATT |  |
| *iucA*-F | GCTTATTTCTCCCCAACCC |  |
| *iucA*-R | TCAGCCCTTTAGCGACAAG |  |

**
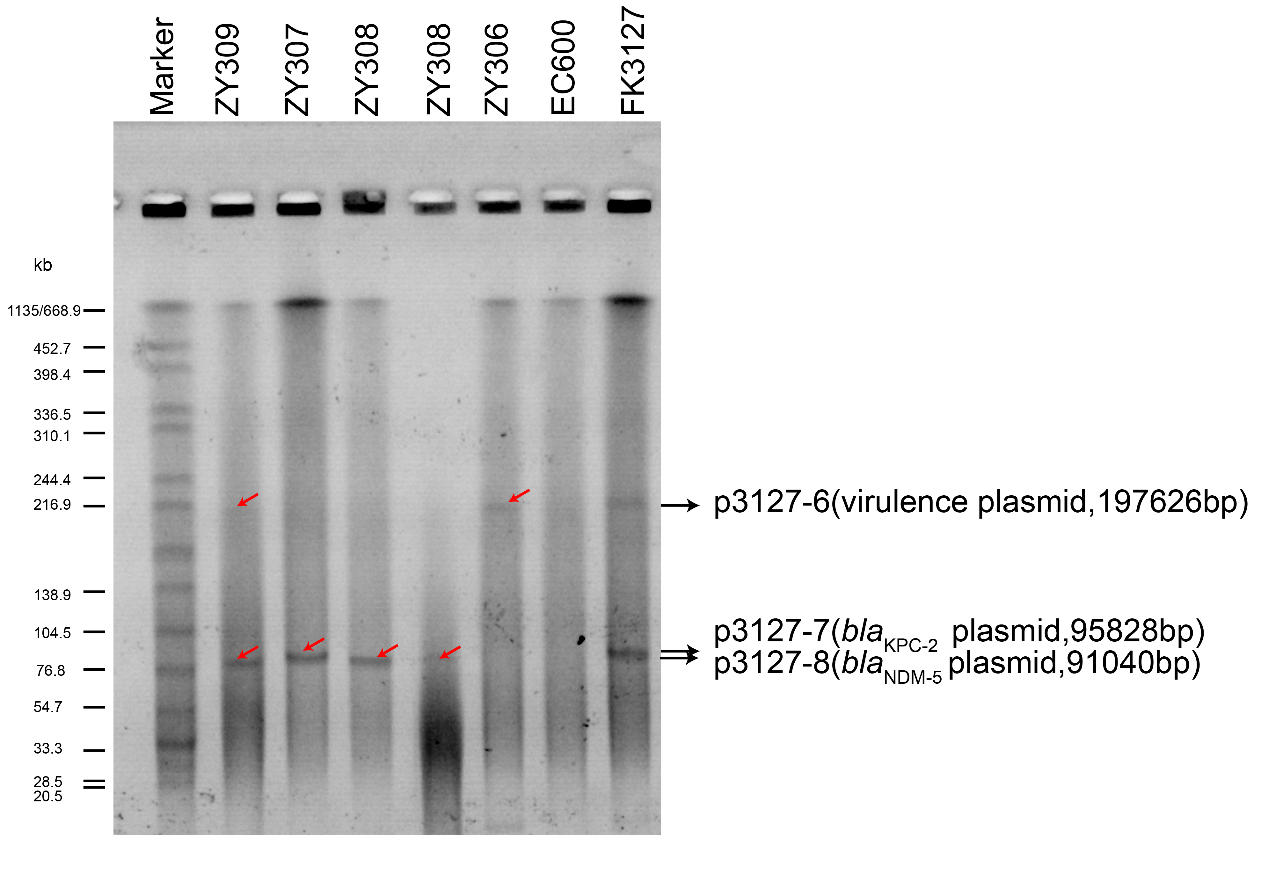
**

**Figure S1. SI-PFGE profiles of original *K. pneumoniae* FK3127 and its and transconjugants.** Lane marker was XbaI-digested DNA of Salmonella Braenderup H9812; Lane FK3127 and Lane EC600 were used as positive reference and negative control, respectively; Transconjugants: ZY306(p3127-6-EC600, virulence), ZY307(p3127-7-EC600, *bla*_KPC-2_), ZY308(p3127-8-EC600, *bla*_NDM-5_), ZY309(p3127-6 & p3127-8-EC600, virulence&*bla*_NDM-5_).
